# Inducible CYP2J2 and Its Product 11,12-EET Promotes Bacterial Phagocytosis: A Role for CYP2J2 Deficiency in the Pathogenesis of Crohn’s Disease?

**DOI:** 10.1371/journal.pone.0075107

**Published:** 2013-09-13

**Authors:** Jonas Bystrom, Scott J. Thomson, Jörgen Johansson, Matthew L. Edin, Darryl C. Zeldin, Derek W. Gilroy, Andrew M. Smith, David Bishop-Bailey

**Affiliations:** 1 William Harvey Research Institute, Queen Mary University, London, United Kingdom; 2 Comparative Biomedical Sciences, Royal Veterinary College, London, United Kingdom; 3 Department of Molecular Biology, Umeå University, Umeå, Sweden; 4 Division of Intramural Research, National Institute of Environmental Health Sciences/National Institutes of Health, Research Triangle Park, North Carolina, United States of America; 5 Department of Medicine, University College London, London, United Kingdom; McMaster University, Canada

## Abstract

The epoxygenase CYP2J2 has an emerging role in inflammation and vascular biology. The role of CYP2J2 in phagocytosis is not known and its regulation in human inflammatory diseases is poorly understood. Here we investigated the role of CYP2J2 in bacterial phagocytosis and its expression in monocytes from healthy controls and Crohns disease patients. CYP2J2 is anti-inflammatory in human peripheral blood monocytes. Bacterial LPS induced CYP2J2 mRNA and protein. The CYP2J2 arachidonic acid products 11,12-EET and 14,15-EET inhibited LPS induced TNFα release. THP-1 monocytes were transformed into macrophages by 48h incubation with phorbol 12-myristate 13-acetate. Epoxygenase inhibition using a non-selective inhibitor SKF525A or a selective CYP2J2 inhibitor Compound 4, inhibited *E. coli* particle phagocytosis, which could be specifically reversed by 11,12-EET. Moreover, epoxygenase inhibition reduced the expression of phagocytosis receptors CD11b and CD68. CD11b also mediates *L. monocytogenes* phagocytosis. Similar, to *E. coli* bioparticle phagocytosis, epoxygenase inhibition also reduced intracellular levels of *L. monocytogenes*, which could be reversed by co-incubation with 11,12-EET. Disrupted bacterial clearance is a hallmark of Crohn’s disease. Unlike macrophages from control donors, macrophages from Crohn’s disease patients showed no induction of CYP2J2 in response to *E. coli*. These results demonstrate that CYP2J2 mediates bacterial phagocytosis in macrophages, and implicates a defect in the CYP2J2 pathway may regulate bacterial clearance in Crohn’s disease.

## Introduction

Monocyte-derived macrophages play a critical role in host defence, wound healing and chronic inflammation [1]. Arachidonic acid is metabolised into families of biologically active mediators by cyclooxygenase, lipoxygenase and CYP450 pathways [2], [3]. The main arachidonic acid-metabolising CYPs are the CYP2 family, [3]–[6], of which CYP2J2 and CYP2C8 are present in human monocytes and macrophages [7]. Recombinant CYP2J2 metabolises arachidonic acid in to all four *cis*-EETs 5,6-EET, 8,9-EET, 11,12-EET and 14,15-EET [8], and all are produced by human macrophages [9]. We recently published that CYP2J2 and its anti-inflammatory products are ligands for the peroxisome-proliferator activated receptor (PPAR) class of nuclear receptors [10]. Moreover, we showed that endogenous epoxygenases are anti-inflammatory in human monocytes and M1 macrophages in part via activation of PPARα. EETs are rapidly metabolized in the body. The main pathway for EET removal is through their conversion into dihydroxyeicosatrienoic acid (DHETs) by soluble epoxide hydrolase (sEH) [11]. DHETs are generally considered to be less active than EETs; however they have been shown to inhibit monocyte migration [12]. Endotoxin-induced lung inflammation is reduced with global sEH knockout [13], which showed significantly reduced activation of e-selectin mRNA, NFκB signaling, and neutrophil infiltration [13]. In addition, sEH knockout or sEH inhibitors reduce the chronic inflammatory bowel disease [14] and its associated tumor formation [15] in IL-10 knockout mice, which was also associated with a reduction in TNFα, MCP-1 and neutrophil infiltration [14]. The roles of CYP450 pathways in mediating responses to pathogens remain poorly understood.

Crohn’s disease is characterized by defects in bacterial clearance [16] and has been associated with an increased burden of bacteria including *E. coli* and *L. monocytogenes* [17]. Here we show CYP2J2 is induced by bacterial stimulation, but is absent in Crohn’s disease macrophages. Moreover, we show CYP2J2 and 11,12-EET mediate the phagocytosis and uptake of *E. coli* and *L. monocytogenes* in macrophages.

## Materials and Methods

### Ethics Statement

Monocyte studies were approved by the Joint University College London (UCL)/UCL Hospitals (UCLH) Committee for the Ethics of Human Research (project numbers 02/0324 and 04/Q0502/29) and conducted according to the Declaration of Helsinki. All volunteers gave written informed consent prior to entering the study.

### Materials

Rabbit polyclonal anti-CYP2J2 was from Abcam (Cambridge, UK). EETs were from Cayman Chemical Company (Cambridge Bioscience, Cambridge, UK). SKF525A was from Biomol (Affiniti Research Products, Exeter, UK). The CYP2J2 inhibitor compound 4 was a gift from Dr Patrick Dansette (Université Paris Descartes, Paris, France; [18]). Taqman primers and reagents and pHrodo Red *E. coli* bioparticles were from Invitrogen (Paisley, Renfrewshire, UK). The human TNFα ELISA was from eBioscience (Hatfield, UK). Unless stated, all other reagents were from Sigma-Aldrich (Poole, Dorset, UK).

### Cell and Tissue Culture

THP-1 were cultured in RPMI supplemented with antibiotic/antimycotic mix, and 10% FBS; 37°C; 5% CO_2_; 95% air. Primary monocytes were isolated from peripheral blood of human volunteers as previously described [19]. The MTT cell viability assay was as previously described [20]. Crohn’s disease macrophages and macrophages from healthy controls were isolated as previously described [16]. Briefly, all patients had definitive diagnoses of Crohn’s disease, confirmed using standard diagnostic criteria, with quiescent disease (Harvey-Bradshaw or Mayo score <3; [21], [22]). Patients on either no medication or a stable maintenance dose of 5-aminosalicylates (2.5 g/d) for the previous 3 months were included. None of the patients had received corticosteroid, immunosuppressant, anti-TNF, or metronidazole therapy within 3 months of enrollment. Healthy control subjects approximately matched for age, sex, and smoking history were recruited. Written informed consent was obtained from all volunteers. Unlike THP-1 cells which can be differentiated in to a macrophage phenotype over 24–48 h by PMA incubation, primary macrophages were produced by culture for 5d. Adherent cells were scraped on day 5 and re-plated in 96-well culture plates at 10^5^/well in X-Vivo-15 medium (Cambrex). Primary monocyte-derived macrophages were incubated overnight to adhere where they were then stimulated with 2.5×10^5^ heat killed *E. coli* for 4 h, prepared as previously described [23].

### RT-PCR

CYP2J2 was measured by Taqman qRT-PCR ddCt method and normalized to GAPDH levels. mRNA expression of phagocytosis receptors was assessed by the Sybr Green ddCT method. Briefly, RNA was extracted using an RNA extraction kit (Thermo Scientific) and 1 µg was used to generate cDNA using Superscript II (Invitrogen) according to manufacturer’s instructions. Sybr green qPCR was performed using Premix Ex Taq II mastermix (Takara) using a Corbett Rotor-Gene 6000 machine. Sequences of primers pairs are listed in Table S1.

### Western Blotting and Immunoassays

CYP2J2 and β-actin protein levels were measured as previously described [8], [24]. TNFα.

ELISA was performed according to manufacturer’s instructions.

### Phagocytosis Assays

THP-1 cells were differentiated in to macrophages over 48 h using 100 nM PMA in RPMI supplemented with 10% fetal bovine calf serum and 50 µg/mL of penicillin and streptomycin. The cells were rested for 24 h. Cells were then treated with epoxygenase inhibitors and/or epoxygenase products for 24 h before addition of the phagophores. pHrodo red *E.coli* BioParticles (1 mg/ml) were added for 2 h, and particle uptake analyzed using a Nikon TE2000 inverted florescent microscope connected to a SPOT-RT digital camera and a FLUO star Galaxy plate reader (BMG Labtech, Germany). For *L. monocytogenes* assays, overnight cultures of *L. monocytogenes* EGDe [25] or EGD/pNF8 (GFP-expressing *L. monocytogenes*) [26] were initiated in LB the day before macrophage infection. The morning of the experiment cultures were serially diluted and grown for an additional 3–4 h. Optical density was determined at 600 nm and cultures were selected and diluted in RPMI to correspond to less than one bacterial particle per macrophage. The bacteria was applied to the THP-1 macrophages, the plates were spun at 2000 rpm for five minutes and left in a 37°C incubator with 5% CO_2_ for 3 or 7 h. Macrophages were washed three times in PBS and subsequently lysed using dH_2_O with 0.2% Tween 20. The macrophage lysates were serially diluted and plated on agar plates and left at 37°C overnight. Colonies were then counted and expressed as % uptake based on initial number of bacteria used to infect the macrophages. Uptake of EGD/pNF8 was analyzed using a Nikon TE2000 inverted florescent microscope connected to a SPOT-RT digital camera, and levels of GFP assessed by analysis using ImageJ software.

## Results

### CYP2J2 is Induced in hPBMCs by LPS

**Figure 1:**
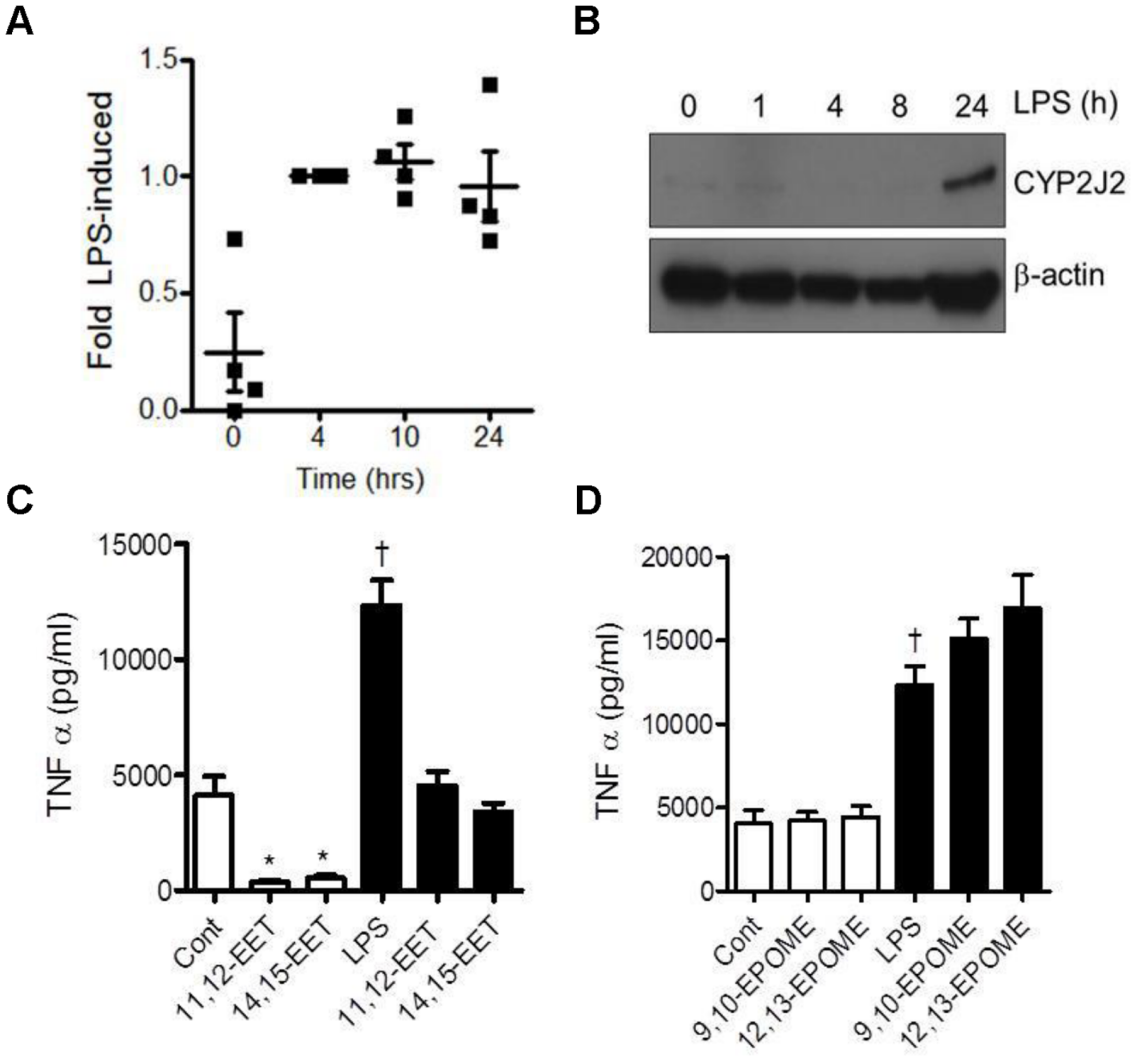
CYP2J2 is an LPS responsive gene: CYP2J2 products feedback to inhibit TNFα release. Time course of CYP2J2 mRNA (A) and protein (B) in human peripheral blood mononuclear cells treated with LPS (10 µg/ml). CYP2J2 mRNA was measured by Taqman RT-PCR and expressed as fold of the LPS induced CYP2J2 expression at 4 h. Data shows data points and mean±s.e.m from 4 individual donors. CYP2J2 protein determined by Western blot was compared to that of β-actin. This data is representative of n = 4 separate donors. (C) Effect of 11,12-EET, or 14,15-EET and (D) 9,10-EPOME, or 12,13-EPOME, on basal and LPS (10 µg/ml; 7 h) induced TNFα release from THP-1 monocytes. TNFα release (pg/ml) in the supernatant was measured by ELISA. The data presents the mean±s.e.m. of n = 8 replicates from 3 separate experiments. *denotes p<0.05 control or LPS and EET treatments, and † denotes p<0.05 between control and LPS, by one-way ANOVA and Bonferroni’s post test.

Treatment of hPBMCs with 10 µg/ml LPS induces CYP2J2 mRNA by 4 h which was still evident by 24 h (Figure 1A), and protein by 24 h (Figure 1B). LPS also induces TNFα release from monocytes (Figures 1C and D), and treatment with epoxygenase arachidonic acid products 11,12-EET and 14,15-EET (1 µM) abolished basal and LPS-induced TNFα release (Figure 1C). In contrast, to the arachidonic acid metabolite EETs, co-treatment with epoxygenase linoleic acid products 9,10-EPOME and 12,13-EPOME (1 µM) had no effect on basal or LPS-induced TNFα release (Figure 1C).

### CYP2J2 Promotes Bacterial Phagocytosis

**Figure 2:**
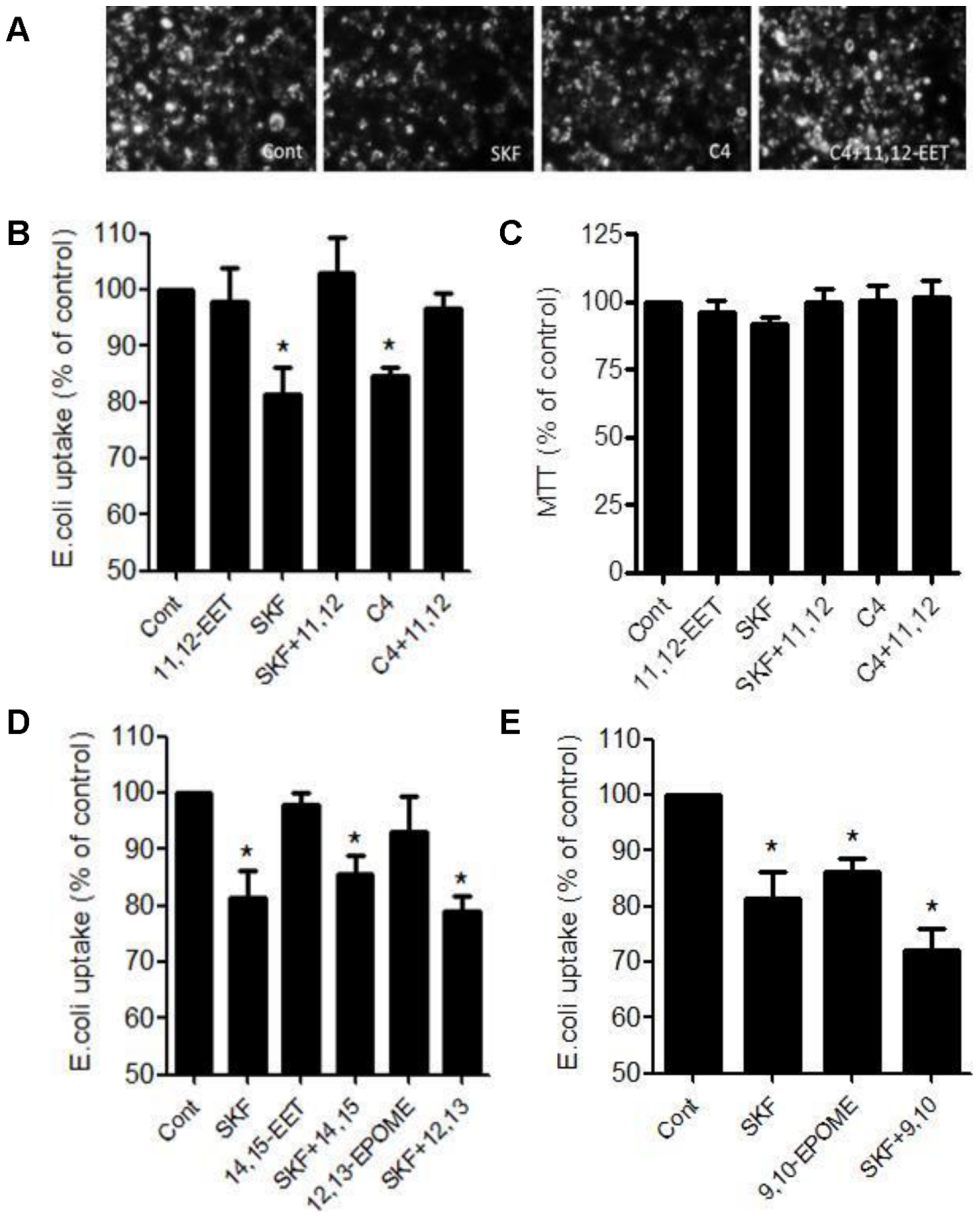
CYP2J2 regulates *E. coli* phagocytosis. (A) Top panel shows representative florescent micrographs and (B) (D), and (E) florescent plate reader recordings of *E. coli* fluorescent bioparticle uptake (1 mg/ml; 2 h) in THP-1 derived macrophages. THP-1 derived macrophages were induced by PMA (100 nM; 48 h). (A) 11,12-EET (1 µM), but not (D) 14,15-EET (1 µM) or 12,13-EPOME (1 µM) reverses SKF525A (10 µM) or Compound 4 (3 µM) reduced *E. coli* particle uptake. (C) MTT viability assay: SKF525A or Compound 4 alone or in combination with 11,12-EET has no effect on cell viability. (E) 9,10-EPOME by itself reduces *E. coli* particle, and acts in an additive manner with SKF25A to reduce *E. coli* particle phagocytosis. Compounds were given as a 24 h pretreatment before addition of *E. coli* bioparticles (1 mg/ml; 2 h). Data represents mean±s.e.m. as a % of control from n = 3 separate experiments. * denotes p<0.05 between control and treatments by one-sample t-test.

THP-1-macrophages rapidly phagocytosed *E. coli* bioparticles after 2 h. 24 h pretreatment with a non-selective epoxygenase inhibitor SKF525A (10 µM) or a selective CYP2J2 inhibitor Compound 4 (3 µM), inhibited *E. coli* bioparticle phagocytosis, which could be reversed by 11,12-EET (1 µM) (Figure 2A and B), but not 14,15-EET (1 µM; Figure 2D). Compound 4 is a high-affinity, competitive inhibitor and alternative substrate of CYP2J2 based upon the structure of terfenadine, which CYP2J2 is known to metabolize [18], [27]. The ability of compound 4 to inhibit CYP2J2 was tested in house by its ability to inhibit CYP2J2 mediated activation of PPARα ([10]; Figure S1). SKF525A, Compound 4 and 11,12-EET had no effect on cell viability in any combination (Figure 2C). Interestingly, 9,10-EPOME (1 µM; Figure 2E), but not 12,13-EPOME (1 µM; Figure 2D) also reduced *E. coli* bioparticle phagocyosis. Treatment with SKF525A, further reduced 9,10-EPOME inhibition of phagocytosis in an additive manner (Figure 2E).

**Figure 3:**
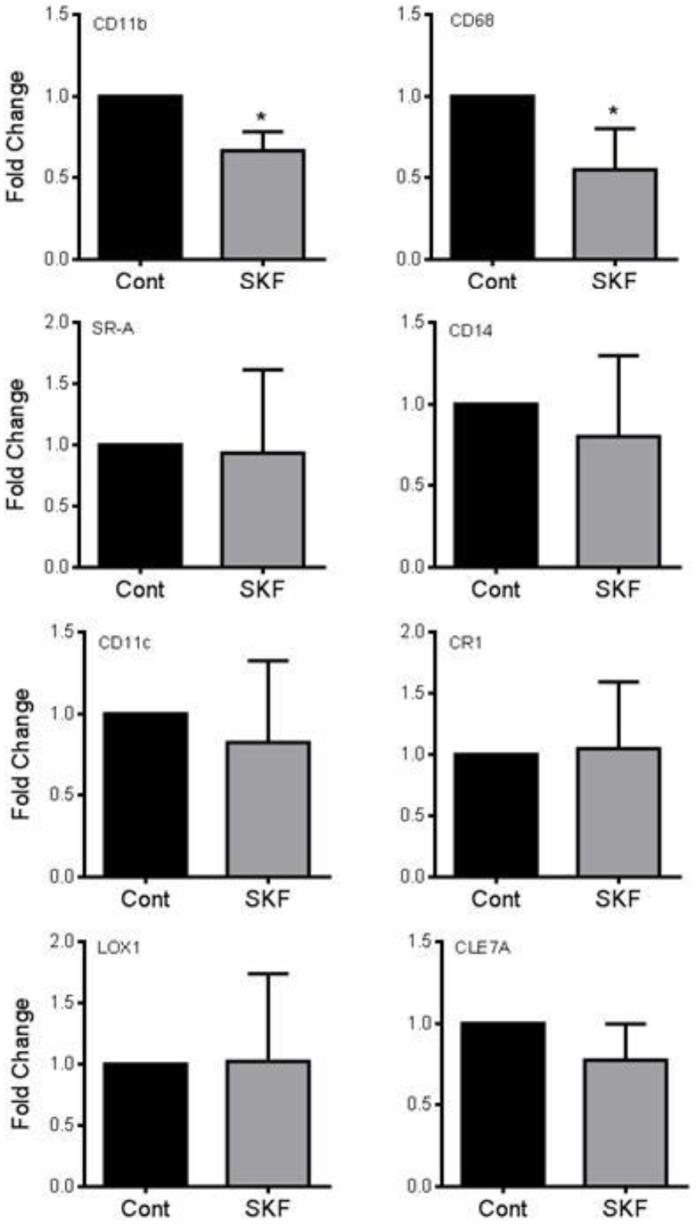
Epoxygenase inhibition regulates CD11b and CD68 phagocytosis receptor expression. Epoxygenase inhibition (SKF525A; 10 µM) reduces expression of CD11b and CD68 mRNA in THP-1–derived macrophages. Levels SR-A, CD14, CD11c, CR1, LOX1 and CLEC7A were unchanged in the presence of SKF525A. Data represents mean±s.e.m. fold change in expression from control, from n = 3–4 separate experiments. * denotes p<0.05 between control and SKF525A by one-sample t-test.

We performed an initial broad spectrum screen of phagocytosis receptors using standard RT-PCR and found SR-B, HPRT, CD11b, CD14, CD68, CD200R, CLEC7A, TIMD4, and CR1, mRNA were detected in THP-1 cells in culture (data not shown). After PMA differentiation in to macrophages, SR-A, CD11b, CD14, LOX1, CLEC7A, CD18 and CD11c were induced, SR-B, CD200R inhibited, and CD68 and HPRT levels remained unchanged (unpublished observations). qRT-PCR analysis of THP-1 macrophages showed the epoxygenase inhibitor SKF525A (10 µM; 24 h) reduced CD11b and CD68 mRNA levels, while levels of SR-A, CD14, CD11c, CR1, LOX1 and CLEC7A remained unchanged (Figure 3).

**Figure 4:**
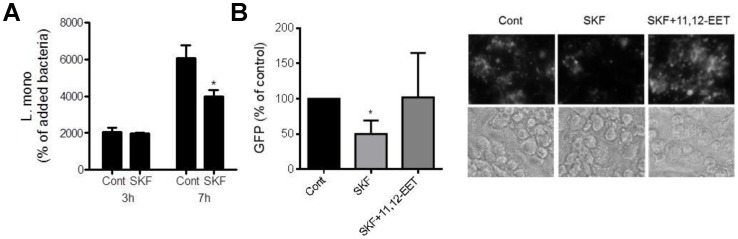
CYP2J2 regulates *L. monocytogenes* phagocytosis. (A) Epoxygenase inhibition (SKF525A 10 µM; SKF; 24 h) inhibits the infection of *L. monocytogenes* into THP-1 derived macrophages, expressed as % of bacteria added at 0 h. (B) Right panel shows representative fluorescent micrographs and left panel image analysis of GFP*-L. monocytogenes* in THP-1 derived macrophages at 7 h. THP-1 derived macrophages were induced by PMA (100 nM; 48 h). 11,12-EET (1 µM) reversed the SKF525A (10 µM) mediated reduction in *L. monocytogenes* infection. Compounds were given as a 24 h pretreatment before addition of *L. monocytogenes*. Data represents mean±s.e.m. as a % of control from n = 3–6 separate experiments. * denotes p<0.05 between control and treatments by paired t-test (A) or one-sample t-test (B).

As well as mediating Gram negative *E. coli* phagocytosis [28], CD11b is known to mediate Gram positive *L. monocytogenes* phagocytosis [29]. SKF525A (10 µM; 24 h) pretreatment reduced the intracellular levels of *L. monocytogenes* (Figure 4A) and GFP-*L. monocytogenes* (Figure 4B). 11,12-EET restored the uptake of GFP-*L. monocytogenes* inhibited by SKF525A (Figure 4B), but was not significant from control by itself (GFP expression; data not shown).

### Crohn’s Disease Macrophages Do not Induce CYP2J2 in Response to E Coli

**Figure 5:**
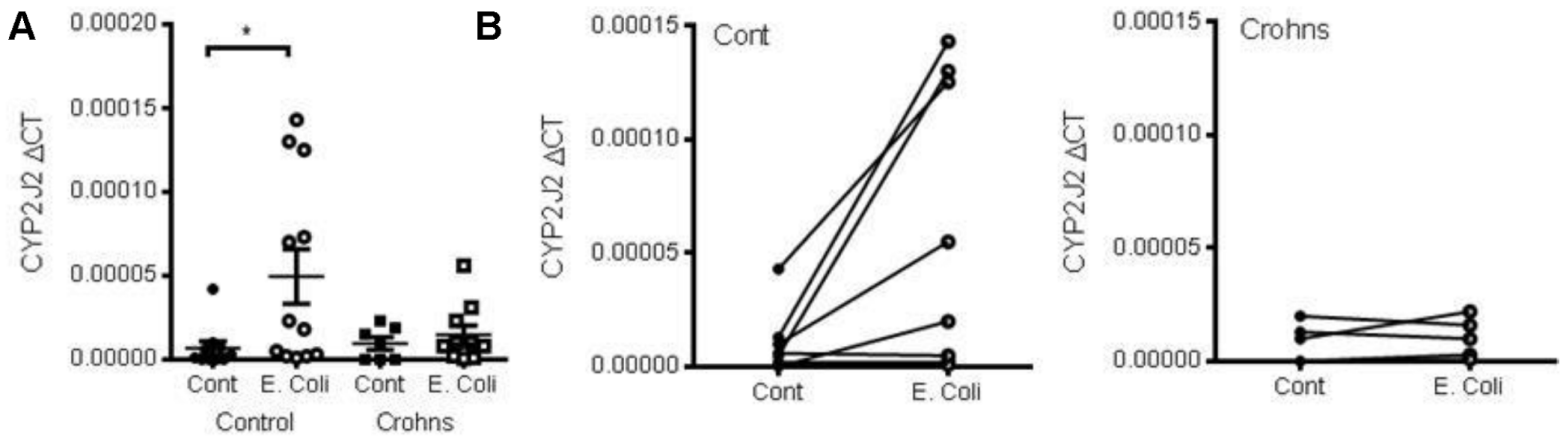
Crohn’s disease macrophages do not induce CYP2J2 in response to *E. coli* stimulation. (A) unpaired analysis and (B) paired analysis of CYP2J2 mRNA induction in macrophages from Crohn’s disease patients and matched controls in response to heat killed *E. coli* (2.5×10^5^; 4 h). CYP2J2 mRNA was measured by Taqman RT-PCR and expressed as dCT (4 h). Data shows data points and mean±s.e.m from 7–13 individual donors (A) or 7 paired control and 5 paired Crohn’s disease samples (B). *denotes p<0.05 between control and *E. coli* treatment, by Mann-Whitney U test.

PBMCs from Crohn’s disease patients and healthy volunteers were differentiated in to macrophages over 5 days. Macrophages were treated with heat inactivated *E. coli* for 4 h. Similar to LPS-treated monocytes (Figure 1), heat-treated *E. coli* induced CYP2J2 expression (Figure 5) in macrophages from healthy controls. In contrast macrophages derived from Crohn’s disease patients showed no induction of CYP2J2 with heat-treated *E. coli* (Figure 5). Figure 5A shows the induction of CYP2J2 by heat-treated *E. coli* in macrophages from healthy volunteer but not Crohn’s disease patients, by an unpaired analysis of 7–13 donors. We did not have paired control and *E. coli* samples for all the donors, so Figure 5B shows the paired analysis from healthy volunteers (n = 7) and Crohn’s disease patients (n = 5) where we did obtain matched control and *E. coli* treatment (Figure 5B).

## Discussion

Here we show CYP2J2 is a LPS/*E. coli* inducible enzyme in PBMCs and macrophages. The CYP2J2 promoter does not contain a TATA box [30], and as such it has not been considered an inflammation or TLR-4 inducible target. However, consistent with these findings, CYP2J2 is up-regulated in preeclampsia and is induced in a trophoblast cell line with TNFα [31]. As reported by us and others, [7], [32], circulating PBMCs contain low levels of CYP2J2. However, similar to TLR-4 stimulation CYP2J2 could also be induced by PMA, M-CSF and GM-CSF [32]. These results together clearly suggest that CYP2J2 is inducible and therefore may be the major inflammation regulated epoxygenase in man.

THP-1 monocytes and M1 macrophages contain CYP2J2 and CYP2C8 [7]. Inhibition of epoxygenases with the non-selective epoxygenase inhibitor SKF525A and a selective chemically distinct CYP2J2 inhibitor, Compound 4 [18] equally reduced *E. coli* phagocytosis, strongly implicating CYP2J2 as the enzyme responsible. We routinely use SKF525A to inhibit CYP2J2 as we previously demonstrated 10 µM SKF (the concentration used in this study) abolishes CYP2J2-dependent activation of PPAR responses [32]. Compound 4 similarly inhibits CYP2J2-dependent activation of peroxisome-proliferator activated receptor responses (Figure S1). *E. coli* uptake was reversed selectively by 11,12-EET, but not 14,15-EET, or linoleic acid epoxygenase products. Indeed, 9,10-EPOME itself reduced phagocytosis. This *E. coli* phagocytosis assay therefore distinguishes 11,12-EET from 14,15-EET and 9,10-EPOME from 12,13-EPOME.

In contrast, 11,12- and 14,15-EET both reduced TNFα release from LPS-stimulated monocytes, consistent with their known anti-inflammatory actions [33]. In contrast the linoleic acid CYP2J2 products 9,10-EPOME and 12,13-EPOME had no effect on TNFα release. As the receptors for epoxygenase products are poorly defined [33] these findings are of particular interest, as the EETs and EPOMEs used here show clear and distinct regio-isomer specific action on phagocytosis and TNFα release, suggesting that specific receptor targets are present and in the future could be identified from these assays.

To examine the potential mechanism by which CYP2J2 could regulate phagocytosis we screened a broad spectrum of phagocytosis receptors by RT-PCR. Of those tested, only CD11b (Mac-1, CR3) and CD68 showed regulation by epoxygenase inhibition, and both were reduced. As CD68 is considered an oxidized lipid receptor and not a bacterial sensing receptor [34], we focused on CD11b. CD11b is a versatile pattern recognition receptor that can mediate both Gram negative (e.g. *E. coli*) as well as Gram positive bacterial phagocytosis [35]. For example, CD11b is associated with gram positive *L. monocytogenes* phagocytosis and killing [29]. We therefore tested whether live *L. monocytogenes* uptake by macrophages could also be affected by epoxygenase inhibition. Similar to the *E. coli* bioparticle, intracellular uptake of live *L. monocytogenes* was inhibited by SKF525A and reversed by 11,12-EET.

*L. monocytogenes,* like *E. coli*, has been found at higher levels in Crohn’s disease tissue [17]. Moreover, *L. monocytogenes and E. coli* infection are a side-effect of anti-TNFα therapy in Crohns disease [36]–[38]. CD11b has also been shown to be expressed at lower levels in Crohn’s disease compared to ulcerative colitis [39]. We therefore examined CYP2J2 in Crohn’s disease macrophages.

The rested macrophages from Crohn’s disease patients similar to healthy controls had low levels of CYP2J2. However, unlike healthy controls, CYP2J2 was not induced by further *E. coli* stimulation. Interestingly, these results confirm some of our previous microarray analysis of macrophages from healthy controls, and patients with ulcerative colitis and Crohn’s disease [16]. CYP2J2 was one of the un-validated targets absent specifically in Crohn’s disease (but not ulcerative colitis) macrophages treated with heat inactivated *E. coli*. The mechanism for this lack of induction is currently under investigation. Although CYP2J2 polymorphisms, particularly the 50G-T promoter polymorphism have been associated with cardiovascular disease and hypertension in some populations [40] a polymorphisms of the CYP2J2 pathway has yet to be associated with any other form of inflammatory disorder in man.

The lack of CYP2J2 in response to inflammation may therefore mediate some of the macrophage defects observed in Crohn’s disease. Using epoxygenase/CYP2J2 inhibitors reveals a dysregulation in bacterial clearance which is also a hallmark of Crohn’s disease [16]. Crohn’s disease is strongly associated with defective bacterial handling that is also linked to abnormalities in autophagy pathways [41]. Interestingly, we found epoxygenases may also regulate macrophage autophagy. Treatment of THP-1 macrophages with SKF525A results in LC3-II induction similar to that of a known autophagy inducer rapamycin A (Figure S2) providing a further link between the two processes which relate to a Crohn’s disease phenotype.

11,12-EET reversed the actions of epoxygenase inhibition in all the assays tested. Elevation of epoxygenase products using soluble epoxide hydrolase inhibitors or the use of EET-agonists/mimetics may therefore be a therapeutic avenue to correct in Crohn’s disease. sEH inhibitors attenuate the chronic colitis associated with IL-10 knockout in mice [14], indicating that epoxygenases are protective at least in animal models of bowel inflammation. Since CYP2J2 appears particularly dysregulated, our results suggest EET mimetics and/or sEH inhibitors may be of particular benefit in Crohn’s disease.

In conclusion, we show CYP2J2 is an inflammatory induced epoxygenase that has anti-inflammatory actions, and promotes Gram positive and Gram negative bacteria phagocytosis. An absence of CYP2J2 in Crohn’s disease macrophages in response to bacterial infection may contribute to the pathogenesis of Crohn’s disease, in part via a reduced expression of CD11b. Elevating epoxygenase products or the use of 11,12-EET mimetics may provide useful therapeutic avenues for the treatment of inflammatory bowel diseases and in particular may correct a defect present in Crohn’s disease.

## Supporting Information

Figure S1 **Inhibition of CYP2J2 by compound 4.** HEK293 cells were transfected with a combination of CYP2J2, PPARα and the PPAR luciferase reporter gene pACO.Luc as previously described^1^. The figure shows the reduction in CYP2J2 mediated PPARα activation by increasing concentrations of CYP2J2 inhibitor compound 4. The data represents mean±s.e.m. from n = 3 separate experiments. ^1^Wray JA, *et al.* The epoxygenases CYP2J2 activates the nuclear receptor PPARalpha in vitro and in vivo. PLoS One. 2009 Oct 12;4(10):e7421. doi: 10.1371/journal.pone.0007421.
(DOCX)

Figure S2 **Epoxygenase regulation of the macrophage autophagy marker LC3-II.** THP-1 derived macrophages were treated with rapamycin A (RAPA; 50 µg/ml; positive control for autophagy; 3h), or SKF525A (10 µM; 24 h), in the presence or absence of 3-methyladenine (3 MA; 5 mM; autophagy inhibitor), or 11,12-EET (1 µM). LC3-II lipidylation from LC3-I was determined by Western blotting and densometric analysis of the bands performed using ImageJ image analysis software. The figure shows the relative expression of LC3-II expression as a % of the RAPA induced LC3-II. Data represents mean±s.e.m. from n = 3 separate experiments. Epoxygenase inhibition induces a comparable level of LC3-II expression as RAPA, which is reversed either my co-incubation with the autophagy inhibitor 3 MA or 11,12-EET.
(DOCX)

Table S1 **Primer pairs for phagocytosis receptors.**
(DOCX)
