# Supplementary material for: Inducible CYP2J2 and Its Product 11,12-EET Promotes Bacterial Phagocytosis: A Role for CYP2J2 Deficiency in the Pathogenesis of Crohn’s Disease?
Source: PLoS One. 2013 Sep 13;8(9):e75107. doi: 10.1371/journal.pone.0075107 (PMC3772848; doi:10.1371/journal.pone.0075107)
Supplement: Figure S1 — Inhibition of CYP2J2 by compound 4. HEK293 cells were transfected with a combination of CYP2J2, PPARα and the PPAR luciferase reporter gene pACO.Luc as previously described1. The figure shows the reduction in CYP2J2 mediated PPARα activation by increasing concentrations of CYP2J2 inhibitor compound 4. The data represents mean±s.e.m. from n = 3 separate experiments. 1Wray JA, et al. The epoxygenases CYP2J2 activates the nuclear receptor PPARalpha in vitro and in vivo. PLoS One. 2009 Oct 12;4(10):e7421. doi: 10.1371/journal.pone.0007421. (DOCX) [file pone.0075107.s001.docx]

**Figure S1. Inhibition of CYP2J2 by compound 4.** HEK293 cells were transfected with a combination of CYP2J2, PPARα and the PPAR luciferase reporter gene pACO.Luc as previously described^1^. The figure shows the reduction in CYP2J2 mediated PPARα activation by increasing concentrations of CYP2J2 inhibitor compound 4. The data represents mean±s.e.m. from n=3 separate experiments.

^1^Wray JA, *et al.* . The epoxygenases CYP2J2 activates the nuclear receptor PPARalpha in vitro and in vivo. PLoS One. 2009 Oct 12;4(10):e7421. doi: 10.1371/journal.pone.0007421.
