# Supplementary material for: Inducible CYP2J2 and Its Product 11,12-EET Promotes Bacterial Phagocytosis: A Role for CYP2J2 Deficiency in the Pathogenesis of Crohn’s Disease?
Source: PLoS One. 2013 Sep 13;8(9):e75107. doi: 10.1371/journal.pone.0075107 (PMC3772848; doi:10.1371/journal.pone.0075107)
Supplement: Figure S2 — Epoxygenase regulation of the macrophage autophagy marker LC3-II. THP-1 derived macrophages were treated with rapamycin A (RAPA; 50 µg/ml; positive control for autophagy; 3h), or SKF525A (10 µM; 24 h), in the presence or absence of 3-methyladenine (3 MA; 5 mM; autophagy inhibitor), or 11,12-EET (1 µM). LC3-II lipidylation from LC3-I was determined by Western blotting and densometric analysis of the bands performed using ImageJ image analysis software. The figure shows the relative expression of LC3-II expression as a % of the RAPA induced LC3-II. Data represents mean±s.e.m. from n = 3 separate experiments. Epoxygenase inhibition induces a comparable level of LC3-II expression as RAPA, which is reversed either my co-incubation with the autophagy inhibitor 3 MA or 11,12-EET. (DOCX) [file pone.0075107.s002.docx]

**Figure S2. Epoxygenase regulation of the macrophage autophagy marker LC3-II.** THP-1 derived macrophages were treated with rapamycin A (RAPA; 50μg/ml; positive control for autophagy; 3h), or SKF525A (10μM; 24h), in the presence or absence of 3-methyladenine (3MA; 5mM; autophagy inhibitor), or 11,12-EET (1μM). LC3-II lipidylation from LC3-I was determined by Western blotting and densometric analysis of the bands performed using ImageJ image analysis software. The figure shows the relative expression of LC3-II expression as a % of the RAPA induced LC3-II. Data represents mean±s.e.m. from n=3 separate experiments. Epoxygenase inhibition induces a comparable level of LC3-II expression as RAPA, which is reversed either my co-incubation with the autophagy inhibitor 3MA or 11,12-EET.
