# Supplementary material for: Inducible CYP2J2 and Its Product 11,12-EET Promotes Bacterial Phagocytosis: A Role for CYP2J2 Deficiency in the Pathogenesis of Crohn’s Disease?
Source: PLoS One. 2013 Sep 13;8(9):e75107. doi: 10.1371/journal.pone.0075107 (PMC3772848; doi:10.1371/journal.pone.0075107)
Supplement: Table S1 — Primer pairs for phagocytosis receptors. (DOCX) [file pone.0075107.s003.docx]

**Table S1. Primer pairs for phagocytosis receptors**

| hsHPRT_F | CTT TGC TGA CCT GCT GGA TTA C | |
| --- | --- | --- |
| hsHPRT_R | CCT GAC CAA GGA AAG CAA AGT C | |
| hsCD36_F | AGC TGC TAT GCC GTG GAA ATC | |
| hsCD36_R | CTG GAT AAG CAG GTC TCC AAC T | |
| hsSR-A_F | GTC CAA TAG GTC CTC CGG GT |  |
| hsSR-A_R | CCA CCG ACC AGT CGA ACT TT |  |
| hSR-B_F | CCA AAT CCG GAG CCA AGA GA | |
| hSR-B_R | GTT GAC GAG CCT CTC CCT AC |  |
| hsCD11b_F | GCT TAC TTG GGT TAT GCT GCC |  |
| hsCD11b_R | GAA CAG CAT CAC ACT GCC AC |  |
| hsCD14_F | TGG GAT ATA AGA GGC AGC CGA | |
| hsCD14_R | ATC GTC CAG CTC ACA AGG TT |  |
| hsCD68_F | GCA GCA CAG TGG ACA TTC TC |  |
| hsCD68_R | GAA GCA AAT GCT CAG AGG GC | |
| hsLOX-1_F | GCT GGG CAT GCA ATT ATC CC |  |
| hsLOX-1_R | CAG ATC CAG TCT TGC GGA CA |  |
| hsCD200R_F | GGC CGC TTC AAG CAG TTT AT |  |
| hsCD200R_R | AGG TGT AAC TAA CAC TTG GAG GT | |
| hsMARCO_F | GCT GCG AGG TTT ACA ACC AG |  |
| hsMARCO_R | GCC TGC AGA TTC AGA ACT TGG | |
| hsCLEC7A_F | GGG TAC CAT GGG GGT TCT TT |  |
| hsCLEC7A_R | GCC GAG AAA GGC CTA TCC AA | |
| hsCLEC6A_F | GGA TGT TGC CCA GCT TCT TG |  |
| hsCLEC6A_R | ACC TAG GTG CCA AAA TCT GAC | |
| hsTimd4_F | CCC ATC CTC ACT GCA GAA TC |  |
| hsTimd4_R | TCC ACA TTG ACA CGT GGG AT |  |
| hsCR1_F | AGG ACA GGT GCA GAC GTA AA | |
| hsCR1_R | TCT CCA TTG GTG ATG GTG GG |  |
| hsCD18_F | AAG GCA GCC CAC ACT TTT CT |  |
| hsCD18_R | GTG AAG TTC AGC TTC TGG CAC | |
| hsCD11c_F | TCC TCC TGT TCA CAG CCT TAG |  |
| hsCD11c_R | GTA CAT GTT CCT CCC GCA CT |  |
